# Epidemiology of invasive meningococcal disease in Canada, 2012–2019

**DOI:** 10.14745/ccdr.v48i05a06

**Published:** 2022-05-05

**Authors:** Myriam Saboui, Raymond SW Tsang, Robert MacTavish, Amisha Agarwal, Y Anita Li, Marina I Salvadori, Susan G Squires

**Affiliations:** 1 Centre for Immunisation and Respiratory Infectious Diseases, Public Health Agency of Canada, Ottawa, ON; 2 National Microbiology Laboratory, Public Health Agency of Canada, Winnipeg, MB

**Keywords:** epidemiology, meningococcal, invasive meningococcal disease, Canada

## Abstract

**Background:**

A variety of routine childhood and adolescent meningococcal vaccination programs using monovalent (serogroup C) and quadrivalent (A, C, Y, W) conjugate vaccines have been implemented in Canada since 2002, resulting in a decrease in invasive meningococcal disease (IMD) incidence, particularly in serogroup C. Meningococcal vaccines have also been used for outbreak response, including the multicomponent vaccine serogroup B vaccine. This report describes the epidemiology of IMD in Canada from 2012 to 2019.

**Methods:**

Case data were obtained from the National Enhanced IMD Surveillance System between January 1, 2012 and December 31, 2019. Isolates were sent to the National Microbiology Laboratory for confirmation of serogroup and further studies including phenotype and clonal complex identification.

**Results:**

A total of 983 cases of IMD were reported between 2012 and 2019. Overall, the age-adjusted incidence of IMD from 2012 to 2019 was 0.34 cases per 100,000 population per year when standardized to the Canadian 2011 population age distribution (95% CI: 0.32–0.36). Infants younger than one year of age had the highest average age-specific incidence rate (3.6 cases per 100,000 population per year, 95% CI: 2.8–4.3). The highest age-adjusted incidence rate was associated with serogroup B (0.17 cases per 100, 000 population per year, 95% CI: 0.16–0.19). Prior to 2015, most invasive serogroup W isolates were identified as clonal complex 22 (ST-22 CC) and the increase in serogroup W in Canada in recent years has been associated with the replacement of the endemic ST-22 CC with the hyper-virulent ST-11 CC.

**Conclusion:**

Invasive meningococcal disease is a rare but severe infection in Canada that mostly affects the very young. Serogroup B continues to account for the greatest proportion of disease. Serogroup W associated with ST-11 CC is becoming a growing contributor of disease in all age groups not protected by serogroup W-containing vaccines.

## Introduction

Invasive meningococcal disease (IMD) is a serious illness caused by the bacterium *Neisseria meningitidis* ((1)). High-risk groups for contracting infection include travellers to endemic areas including the sub-Saharan African meningitis belt, young children, adolescents and individuals living in crowded quarters ((2)). Invasive meningococcal disease caused by some serogroups is vaccine preventable and endemic in Canada, with increased activity occurring periodically in the winter months. Since the early 2000s, a variety of routine childhood and adolescent vaccination programs using monovalent (serogroup C) and quadravalent (serogroup A, C, Y and W-135) meningococcal conjugate vaccines have been implemented in Canada ((3). This has resulted in a decrease in IMD incidence, particularly for serogroup C. In the last decade, the multicomponent meningococcal B vaccine (4CMenB) has been used to control outbreaks ((3) but has not been used in routine vaccination programs across the country. During the period covered in this report, the schedule of publicly funded vaccination programs has remained constant.

The last national IMD surveillance report described the epidemiology of IMD in Canada from 2006 to 2011. This article focuses on recent IMD data from 2012 to 2019 and explores trends over time, seasonality, spatial distribution and clinical presentation of cases and mortality.

## Methods

### Data

National enhanced case-based surveillance has been conducted in Canada since 1995 through the Public Health Agency of Canada’s Enhanced Invasive Meningococcal Disease Surveillance System (eIMDSS). Provincial and territorial health departments voluntarily reported non-nominal epidemiologic data on confirmed IMD cases on an annual basis. Deterministic matching was conducted to retrospectively link epidemiologic and laboratory data. This article is based on IMD data extracted from eIMDSS, with disease onset between January 1, 2012, and December 31, 2019. See **Annex** for the national case definition.

### Laboratory methods

Isolates were routinely sent to the National Microbiology Laboratory (NML) for confirmation of serogroup and further strain characterization. Serogrouping was done by bacterial agglutination test and confirmed by polymerase chain reaction (PCR) when necessary; serosubtypes of isolates were determined by whole cell enzyme-linked immunosorbent assay using monoclonal antibodies, while clonal analysis was done by multi-locus sequence typing as previously described ((4)).

### Statistical analysis

The demographic, temporal and spatial distribution of IMD cases were examined, in addition to case manifestation, vaccination history, disease outcome and isolate characteristics. Incidence rates were calculated per 100,000 population using annual July 1^st^ population estimates by province/territory, age and sex, which were obtained from Statistics Canada ((5)). The direct method was used for calculating age-standardized rates based on the 2011 Canadian census. Cases with missing age were excluded from the age standardization and all age-related analyses. Confidence intervals (CIs) for incidence rates were calculated at the 95% confidence level. The CIs of age-standardized rates were calculated according to the method based on the gamma distribution. All analyses were conducted using Microsoft Excel 2010, SAS 9.4, and R version 4.0.2 (R Foundation for Statistical Computing, Vienna, Austria).

## Results

### Trends over time

**Figure 1:**
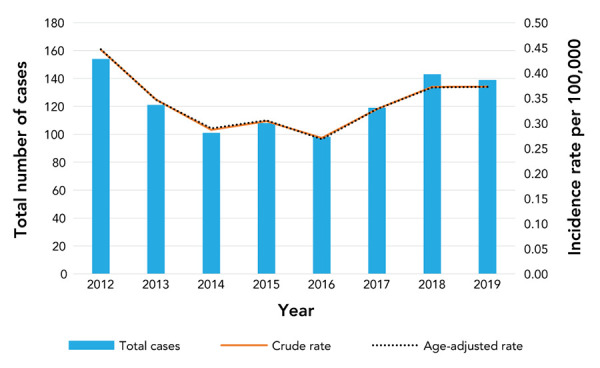
Number of invasive meningococcal disease cases and overall incidence rates (per 100,000 population) in Canada by year, 2012–2019

A total of 983 cases of IMD were reported between 2012 and 2019. Seven cases were excluded from age-specific analyses as the age was missing. The yearly number of reported cases ranged from 98 in 2016 to 154 in 2012 (**Figure 1**). Overall, the age-adjusted incidence of IMD from 2012 to 2019 was 0.34 cases per 100,000 population per year (95% CI: 0.32–0.36). As shown in **Table 1**, those patients 40 years of age and older accounted for 42% of all IMD cases between 2012 and 2019, followed by those 15–19 years (12%), 1–4 years (12%) and younger than one year of age (11%). Among serogroup B cases, children 1–4 years of age accounted for the largest proportion of cases (n=90/487, 18%), although those younger than one year of age and 60 years and older followed closely behind (15% of total cases for each age group). Adults over 40 years of age accounted for the majority of cases associated with serogroups C, W-135 and Y (55%, 56% and 63%, respectively).

**Table 1:** Number and proportion of invasive meningococcal disease cases in Canada for each serogroup by age group, 2012–2019 (N=983 cases)

Age group (years)	Number and proportion of invasive meningococcal disease cases															
	Serogroup								Non-groupable		Other^a^		Unknown^b^		Total	
	B		C		W-135		Y									
	n	%	n	%	n	%	n	%	n	%	n	%	n	%	n	%
<1	73	15	4	7	18	10	5	3	0	0	0	0	9	17	109	11
1–4	90	18	2	4	11	6	3	2	0	0	0	0	9	17	115	12
5–9	18	4	2	4	2	1	2	1	0	0	2	18	2	4	28	3
10–14	20	4	2	4	2	1	3	2	0	0	1	9	2	4	30	3
15–19	69	14	4	7	13	8	26	13	1	25	1	9	6	11	120	12
20–24	36	7	4	7	11	6	19	10	0	0	4	36	3	6	77	8
25–29	21	4	0	0	7	4	6	3	0	0	1	9	2	4	37	4
30–39	23	5	7	13	7	4	8	4	1	25	0	0	4	8	50	5
40–59	62	13	13	23	33	19	44	22	1	25	2	18	10	19	165	17
60+	75	15	18	32	63	37	82	41	1	25	0	0	6	11	245	25
Unknown	0	0	0	0	5	3	2	1	0	0	0	0	0	0	7	1
Total	487	–	56	–	172	–	200	–	4	–	11	–	53	–	983	–

Abbreviation: –, not obtained

^a^ Other are those cases with the serogroup noted as: A, E, Z, 29E and non-encapsulated

^b^ Unknown are those cases missing serogroup information

The highest incidence rate observed was associated with serogroup B in 2012 (0.32 cases per 100,000, 95% CI: 0.26–0.38) (**Table 2**); since then, the rate has declined. The incidence rate for serogroups C and Y has remained low and stable throughout 2012–2019. In more recent years, the incidence rate was highest for serogroup W-135 (0.15 cases per 100,000, 95% CI: 0.11–0.19 in 2018 and 0.12 cases per 100,000, 95% CI: 0.09–0.16 in 2019).

**Table 2:** Age-standardized incidence rates (per 100,000 population) and 95% CI of invasive meningococcal disease in Canada by serogroup and year, 2012–2019 (N=976 cases^a^)

Serogroup	Age-standardized incidence rates (per 100,000 population)															
	2012		2013		2014		2015		2016		2017		2018		2019	
	Mean	95% CI	Mean	95% CI	Mean	95% CI	Mean	95% CI	Mean	95% CI	Mean	95% CI	Mean	95% CI	Mean	95% CI
B	0.32	0.26–0.38	0.23	0.18–0.29	0.16	0.12–0.20	0.18	0.14–0.23	0.13	0.09–0.17	0.14	0.10–0.18	0.12	0.08–0.16	0.12	0.09–0.16
C	0.04	0.02–0.06	0.02	0.01–0.04	0.03	0.01–0.05	0.01	0.0–0.03	0.01	0.0–0.02	0.02	0.01–0.05	0.02	0.01–0.04	0.01	0.0–0.03
W-135	0.01	0.0–0.03	0.01	0.0–0.03	0.02	0.01–0.04	0.03	0.01–0.05	0.04	0.02–0.06	0.08	0.05–0.11	0.15	0.11–0.19	0.12	0.09–0.16
Y	0.05	0.03–0.08	0.07	0.04–0.10	0.08	0.06–0.12	0.07	0.05–0.10	0.07	0.04–0.10	0.06	0.03–0.09	0.07	0.05–0.11	0.08	0.05–0.11
Non-groupable	–	–	–	–	–	–	0.01	0.0–0.02	–	–	–	–	–	–	0.01	0.0–0.02
Other^b^	–	–	–	–	–	–	–	–	0.01	0.0–0.02	0.01	0.0–0.02	–	–	0.01	0.0–0.02
Unknown^c^	0.03	0.01–0.05	0.01	0.0–0.03	0.01	0.0–0.02	0.01	0.0–0.03	0.02	0.01–0.04	0.03	0.01–0.05	0.01	0.01–0.03	0.03	0.0–0.05

Abbreviations: CI, confidence interval, –, not obtained

^a^ Seven cases of total sample were missing age

^b^ Other are those cases with the serogroup noted as: A, E, Z, 29E and non-encapsulated

^c^ Unknown are those cases missing serogroup information

As seen in **Table 3**, there was a statistically significant decrease in the overall age-standardized incidence rates from 2012 to 2016 (approximate decrease of 0.040 cases per year, 95% CI: 0.003–0.077). The opposite was true from 2016 to 2019, where the overall age-standardized incidence rate increased by 0.038 cases per year (95% CI: −0.005–0.076), although this increase was not statistically significant. Infants younger than one year of age had the highest average age-specific incidence rate of 3.6 cases per 100,000 population per year (95% CI: 2.8–4.3), while the incidence ranged from 0.13 to 0.93 per 100,000 population for the other age groups.

**Table 3:** Incidence rates (per 100,000 population) of invasive meningococcal disease in Canada by age group and year, 2012–2019 (N=976 cases^a^)

Age group (years)	Incidence rates (per 100,000 population) by year									
	2012	2013	2014	2015	2016	2017	2018	2019	2012–2019	
									Mean	95% CI
<1	3.42	3.65	4.96	2.86	1.56	3.38	4.20	4.46	3.56	2.83–4.29
1–4	1.18	1.17	0.97	0.91	0.84	0.51	0.83	1.02	0.93	0.78–1.08
5–9	0.44	0.27	0.10	0.05	0.20	0.20	0.05	0.15	0.18	0.09–0.27
10–14	0.68	0.16	0.11	0.21	0.10	0.16	0.05	0.10	0.20	0.06–0.34
15–19	1.17	0.73	0.84	0.66	0.52	0.71	0.47	0.47	0.70	0.54–0.86
20–24	0.51	0.25	0.17	0.33	0.25	0.79	0.37	0.53	0.40	0.26–0.54
25–29	0.25	0.25	0.12	0.12	0.16	0.08	0.12	0.39	0.19	0.12–0.26
30–39	0.13	0.13	0.06	0.15	0.10	0.10	0.16	0.20	0.13	0.10–0.16
40–59	0.31	0.16	0.18	0.20	0.13	0.15	0.31	0.20	0.20	0.15–0.25
60+	0.28	0.42	0.22	0.33	0.41	0.42	0.52	0.42	0.38	0.31–0.44
Overall: crude	0.45	0.35	0.29	0.30	0.27	0.33	0.37	0.37	0.34	0.34
Overall: age-standardized	0.45 (0.38–0.52)	0.35 (0.29–0.41)	0.29 (0.24–0.35)	0.31 (0.25–0.37)	0.27 (0.22–0.33)	0.33 (0.27–0.39)	0.37 (0.31–0.44)	0.37 (0.31–0.44)	0.34 (0.32–0.36)	0.34 (0.32–0.36)

Abbreviation: CI, confidence interval

^a^ N=7 cases of total sample missing age

### Spatial distribution of cases

**Figure 2:**
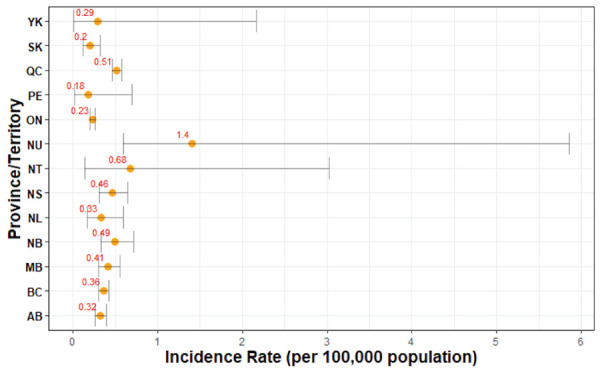
Age-standardized incidence rates (per 100,000 population-year, with 95% CIs) of invasive meningococcal disease in Canada by province or territory, 2012–2019 (N=976 cases) Abbreviations: AB, Alberta; BC, British Columbia; CI, confidence intervals; MB, Manitoba; NB, New Brunswick; NL, Newfoundland and Labrador; NS, Nova Scotia; NT, Northwest Territories; NU, Nunavut; ON, Ontario; QC, Québec; PE, Prince Edward Island; SK, Saskatchewan; YK, Yukon

Between 2012 and 2019, age-standardized incidence rates were highest in the territory of Nunavut (1.40 cases per 100,000 population per year, 95% CI: 0.59–5.86), which was significantly higher than British Columbia, Alberta, Saskatchewan, Manitoba, Québec and Ontario (**Figure 2**); however, only eight cases were reported in Nunavut between 2012 and 2019 resulting in wide 95% CIs. Ontario had the lowest age-standardized incidence rate at 0.23 cases per 100,000 population per year (95% CI: 0.20–0.26), which was significantly lower than Québec, Manitoba, Nova Scotia, New Brunswick, Nunavut and British Columbia. Overall, the provinces and territories had comparable age-standardized incidence rates.

### Travel and seasonality

Between 2012 and 2019, 51 cases out of 376 cases with available data (14%) were associated with travel. Approximately half of the cases (n=27/51, 53%) travelled within Canada. The United States accounted for the largest proportion of cases associated with travel outside the country (n=5/51, 10%).

Data on month of onset were reported for 930 cases (95% of total sample). Overall, the fall and winter months accounted for the majority of cases (60%). Peaks of IMD onset were observed in the months of December/January (22%), March (12%) and October (10%). The summer months (July and August) were characterized by low IMD activity, with only 11% of all cases reported during the summer.

### Clinical presentation and severity

Clinical presentation data were available for 532 samples (51% of total sample), of which 278 cases (52%) presented with meningitis, 182 (34%) with septicemia/bacteremia, 12 (2%) with septicemia/arthritis and 60 (11%) with other manifestations (**Table 4**). Among the 17 cases with “other manifestations” reported, the most frequently reported clinical presentations were fever (35%), pneumonia (24%) and purpura rash (18%). Meningitis was the most common clinical presentation among all serogroups, except for serogroup W-135 where septicemia/bacteremia was most common.

**Table 4:** Number and proportion of invasive meningococcal disease cases in Canada with a clinical presentation by serogroup, (N=503)

Serogroup	Number and proportion of invasive meningococcal disease cases^a^								
	Meningitis		Septicemia/bacteremia		Septicemia/arthritis		Other		Total
	n	%	n	%	n	%	n	%	
B	135	64	53	25	2	1	20	10	210
C	24	52	17	37	3	7	2	2	46
W-135	38	36	43	41	3	3	21	20	105
Y	66	47	57	41	4	3	13	9	140
Other^b^	4	50	4	50	0	0	0	0	8
Non-groupable	0	0	1	100	0	0	0	0	1
Unknown^c^	11	50	7	32	0	0	4	18	22
Total	278	52	182	34	12	2	60	11	532

^a^ 23 cases presented with two diagnoses and three cases presented with three diagnoses

^b^ Other are those cases with the serogroup noted as: A, E, Z, 29E, and non-encapsulated

^c^ Unknown are those cases missing serogroup information

**Figure 3:**
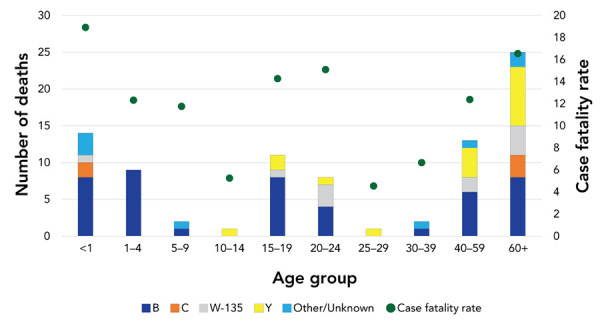
Number of invasive meningococcal disease deaths and case fatality ratio by age group and serogroup, Canada, 2012–2019 (N=621^a^) ^a^ Death (yes/no) only available for 621 cases (63% of total sample). 247 cases (25%) were noted as “unknown” and the rest were missing outcome information (12%)

Between 2012 and 2019, of the 621 cases with outcome data available (yes/no for death), 86 deaths associated with IMD were reported. This represents an overall case fatality rate of 13.8%. As seen in **Figure 3**, serogroup B accounted for the majority (52%) of deaths reported. The case fatality rate was highest in those younger than one year of age (19%), followed closely by those 60 years and over (17%) and those aged 20–24 years of age (15%). The proportion of cases who died from IMD was highest in cases for which the positive specimen was blood (n=68/432, 16%) compared with cerebrospinal fluid specimens (n=15/182, 8%) (difference of 8%, 95% CI 1.8–13.1%). Of the 15 cases whose positive specimen was joint fluid, there were no deaths reported.

## Strain characteristics

### Serogroup distribution of culture-confirmed invasive meningococcal disease

The NML received a total of 772 (79%) case isolates of *N. meningitidis* between 2012 and 2019. Serogroup B was the most common (accounted for 48.9% of all isolates), followed by serogroup Y (23.8%), serogroup W (20.0%) and serogroup C (6.0%), with the remaining 1.3% or 10 isolates identified as serogroup E (three isolates), serogroup Z (two isolates) or non-encapsulated (five isolates). A steady increase in serogroup W was observed over time with 2.7% of the IMD case isolates received at the NML in 2012, to 3.9% in 2013, 4.8% in 2014, 10.1% in 2015, 18.8% in 2016, 27.1% in 2017, 44.7% in 2018 and 39.7% in 2019. By 2018, and into 2019 and 2020 (*unpublished data, data for 2020, NML*) serogroup W was the most encountered serogroup among all IMD isolates received at the NML.

### Serogroup B

No particular clone appeared to predominant in Canada. The most encountered serogroup B clonal complexes (CC) (in order of frequency of occurrence) were sequence type (ST)-269 CC, ST-41/44 CC, ST-213 CC, ST-32 CC, ST-35 CC and ST-1157 CC (each with at least five isolates), which together made up 90% of all the serogroup B IMD isolates from 2012 to 2019. From 2015 to 2019, there were only 189 serogroup B IMD case isolates received at the NML and the most encountered CCs were ST-41/44 CC, ST-269 CC, ST-213 CC, ST-32 CC and ST-1157 CC (each with at least five isolates). Together, they accounted for 84% of all the serogroup B isolates.

### Serogroup W

Prior to 2015, most invasive serogroup W isolates were identified as ST-22 CC and the increase in serogroup W in Canada in recent years was associated with the replacement of the endemic ST-22 CC with the hyper-virulent ST-11 CC ((6)). They were characterized by the antigenic formula of W:2a:P1.5,2 with most typed as ST-11 and of the sub-lineage 11.1 (7).

### Serogroup Y

The ST-23 CC continued to be the most encountered serogroup Y CC, followed by ST-167 CC and ST-174 CC.

### Serogroup C

Of the 49 serogroup C IMD case isolates received at the NML, 36 were typed as ST-11 CC and 13 were typed as non-ST-11 CC. Of the 36 ST-11 CC isolates; 15 were further subtyped as the ET-15 type (sub-lineage 11.2), 20 were non-ET-15 type or sub-lineage 11.1 and one was without further subtyping information.

Non-ST-11 CC serogroup C isolates included three typed as ST-1195 (ST-269 CC) and all three were found in Alberta (in 2017, 2018 and 2019) and had the antigenic formula C:NT:P1.9. There were also two serogroup C isolates typed as ST-4821 (ST-4821 CC) and both were found in British Columbia (in 2013 and 2014) and had the antigenic formula C:NT:P1.1. Additionally, there were three serogroup C isolates typed as ST-35 CC, three typed as ST-103 CC, one typed as ST-41/44 CC and one with a sequence type not assigned to any known CC.

## Discussion

Since the implementation of publicly funded meningococcal C and meningococcal quadrivalent vaccination programs in Canada, the overall incidence of IMD has decreased significantly. Over the surveillance period, vaccination coverage for the meningococcal conjugate C vaccine has remained stable in children under two years of age, ranging from 87.6% to 88.7% between 2013 and 2017 ((8)).

The downward trend in incidence was noted across all Canadian regions as early as the year following implementation of publicly funded programs. Between 2012 and 2019, the Prairies and Central regions reached an all-time low incidence rate. Consistent with the previous surveillance report, Nunavut continued to report the highest incidence rate ((9)). The overall mean incidence in the previously published surveillance report was 0.58 cases per 100,000 population for 2006–2011 compared with 0.34 cases per 100,000 population for 2012–2019 ((6)). Serogroup B accounted for the largest proportion of cases, and disproportionately affected infants under the age of one year. Similar to previously published reports, cases of IMD predominated in the winter months with the highest levels in January and March ((4,10)).

Beginning in 2016, the number of IMD cases associated with serogroup W increased by at least 50% every year in Canada. Between 2012 and 2019, the largest proportion of serogroup W cases associated with clonal complex 11 were reported in adults aged 60 years and older. The emergence of serogroup W cases associated with clonal complex 11 has been reported in previous studies ((10,11)) and our results are consistent with these findings. The NML also reported on the growing number of serogroup W strains in Canada over time ((4,12)). Genomic isolates of the Canadian serogroup W isolates showed the majority of the recent isolates belonged to the South American strain type or sub-lineage, although a small number isolated up to 2016 belonged to the Hajj-type strain ((13)).

During the time period 2012–2019, there were three documented Canadian outbreaks ((14)). One outbreak spanned from 2006 to 2013 in the region of Saguenay-Lac-Saint-Jean, Québec, and was due to serogroup B. A vaccination campaign was launched the following year in the region using the 4CMENB vaccine ((15)). The incidence of IMD due to serogroup B fell dramatically in the targeted population of the vaccination campaign (up to 20 years of age) and in 2015, an outbreak was reported in a university in Nova Scotia ((14)). The first documented outbreak due to serogroup CC-11 was reported in British Columbia in 2017 and resulted in five cases in adolescents aged 15–19 years ((14)).

Antibiotic resistant *N. meningitidis* was recently observed in some serogroup Y isolates that belonged to ST-3587 (ST-23 CC) clone and was characterized by the production of β-lactamase due to presence of the *bla*_ROB-1_ gene likely to be acquired from *Haemophilus influenzae* ((16)). This strain has also been identified in France ((17)) and in the United States, which led to the release of a health alert in June 2020 ((16)). In the United States, this strain has also acquired resistance to ciprofloxacin, an antibiotic commonly used for chemoprophylaxis of close contacts of IMD cases ((18)). Up to now, the expansion and spread of this clone has not been observed in Canada (*unpublished data, NML*).

Laboratory surveillance is important for the identification of novel strains that may contribute to the evolving nature of IMD. For example, the novel South American-United Kingdom strain of ST-11 serogroup W has been responsible for the increase in meningococcal W disease globally and has led to reports of unusual clinical presentations ((7,19,20)). The recent increase in meningococcal C disease in the African meningitis belt was due to a new strain that arose when a ST-10217 non-encapsulated strain from a healthy carrier in Burkina Faso acquired a serogroup C capsule ((21)). The recent emergence in the United States of β-lactamase positive serogroup Y *N. meningitidis* with additional resistance to fluoroquinolones has important implications for treatment and chemoprophylaxis of IMD ((22)).

Imported cases via travel accounted for a small proportion of cases, and the majority of cases due to travel were within Canada or the United States. Laboratory characterization of strains identified two potential imported cases with unusual characteristics of C:NT:P1.14, ST-4821 and resistance to ciprofloxacin. These strain characteristics have not been found in any Canadian isolates previously but were commonly associated with a unique serogroup C clone in China ((4)), which suggested that these two isolates were from imported cases.

Serogroup B accounted for the largest proportions of clinical presentations. Between 2012 and 2019, the mortality rate associated with IMD continued to decline over time and serogroup B accounted for the majority of deaths. The trends observed in this report are consistent with evidence from other publications ((6)). Mortality rates in Canada were comparable to those of Australia and England ((7,13)). Positive specimens from blood resulted in nearly double the mortality ratio when compared with positive specimens from cerebrospinal fluid. This finding was statistically significant.

In recent years, evidence of atypical clinical presentation of IMD serogroup W characterized by gastrointestinal symptoms has been reported across the world ((23–26)). Among the cases reported with other clinical manifestations in Canada between 2012 and 2019, the majority were associated with serogroup W-135. Heightened surveillance of atypical presentations in Canada is recommended to better understand the evolving epidemiology of IMD.

The World Health Organization established a plan to defeat meningitis by 2030 ((27)). The plan includes three main goals pertaining to epidemics, disease transmission and disabilities and long-term effects of infection: 1) the elimination of IMD epidemics; 2) the reduction of cases and deaths from vaccine preventable bacterial meningitis; and 3) the reduction of disability associated with infections. Although IMD remains endemic in Canada, evidence from this article shows noteworthy reductions in incidence rates in some geographic areas in Canada. The introduction of publicly funded vaccination programs in Canada has sharply decreased the incidence of IMD associated with strains covered by the vaccines. New strains of IMD will continue to pose challenges for disease target reductions such as the ones outlined by the World Health Organization. Canada has a strong public health infrastructure that allows for early detection of cases and the implementation of prompt public health actions; however, the current routine surveillance at the national level does not collect data on cases post-infection, such as long-term disabilities.

### Limitations

Several limitations should be noted. First, data reported to the eIMDSS surveillance system are not epidemiologically linked with laboratory data. To connect the two data sources, deterministic matching was done. Some cases may have been missed due to insufficient data to match the two data sources. Second, many cases might not be recognized, as antibiotics are frequently given prior to taking cultures and the organism would not be identified. Third, certain data variables, such as immunization history, were often missing or incomplete. Finally, limited outbreak information represents a gap in national IMD surveillance.

### Conclusion

Invasive meningococcal disease is a rare but severe infection that continues to affect the very young population in Canada. While serogroup B continues to account for the greatest proportion of disease, serogroup W associated with CC-11 is becoming a growing contributor of disease in older adults. The evolving nature of *N. meningitidis* requires a comprehensive approach to its surveillance, which should include a laboratory component that documents strain characteristics.

## Annex: National case definition of invasive meningococcal disease as of November 2009

### Confirmed case

Clinical evidence of invasive disease with laboratory confirmation of infection:

Isolation of *Neisseria meningitidis* (*N. meningitidis*) from a normally sterile site (blood, cerebrospinal fluid (CSF], joint, pleural or pericardial fluid)

OR

Demonstration of *N. meningitidis* DNA by an appropriately validated nucleic acid test (NAT) from a normally sterile site

### Probable case

Clinical evidence of invasive disease with purpura fulminans or petechiae, with no other apparent cause and with non-confirmatory laboratory evidence:

Detection of *N. meningitidis* antigen in the CSF

Note: Positive antigen test results from urine and serum samples are unreliable for diagnosing meningococcal disease.

### Clinical evidence

Clinical illness associated with invasive meningococcal disease usually manifests itself as meningitis and/or septicaemia, although other manifestations may be observed (e.g. orbital cellulitis, septic arthritis). Invasive disease may progress rapidly to petechiae or purpura fulminans, shock and death. Both confirmed and probable cases of disease should be notified as of January 1, 2006.
